# Evaluations of the *in vivo* laxative effects of aqueous root extracts of *Euclea racemosa* L. in mice

**DOI:** 10.1016/j.metop.2022.100222

**Published:** 2022-12-17

**Authors:** Akeberegn Gorems Ayele, Brooktawit Mulugeta, Yohannes Tsegyie Wondmkun

**Affiliations:** Department of Pharmacology and Clinical Pharmacy, School of Pharmacy, College of Health Sciences, Addis Ababa University, Addis Ababa, Ethiopia

**Keywords:** Laxative, Constipation, *E. racemosa* L., Loperamide, Motility, CFTR, Cystic fibrosis transmembrane conductance regulator, *E. racemosa* L., *Euclea racemosa* L., GI, Gastrointestinal

## Abstract

**Background:**

Management of constipation with currently available modern medicines is costly and chances of side effects are high. This limits their clinical usefulness and remain to be solved, and calls for investigations of new and better compounds. The experimental plant, *Euclea racemosa* L. *(E. racemosa* L) is among plants, which are used for management of constipation traditionally but its effect is not yet experimentally validated. Therefore, the aim of the present study is to investigate the laxative effects of this plant.

**Methods:**

The laxative effects of aqueous root extracts of *E. racemosa* L. were evaluated using gastrointestinal motility, laxative activity, and gastrointestinal secretion tests.

**Results:**

In the laxative test, the 200 and 400 mg/kg doses of plant extract showed a significant increase in percent fecal water content. The plant extract also significantly accelerated the charcoal meal in gastrointestinal motility test of loperamide-constipated mice. Moreover, the experimental plant produced significant Gastrointestinal (GI) transit ratio at all doses but failed to produce a significantly higher fluid accumulation except 400 mg/kg doses of extract in gastrointestinal secretion test. The observed effect of the aqueous root extract might be due to the presence of secondary metabolites. The aqueous root extract of *E. racemosa* L. revealed the presence of terpenes, saponins, flavonoids and phenols when it was subjected to phytochemical screening*.*

**Conclusion:**

The investigation obtained from this study suggested that *E. racemosa* L. has a beneficial effect in producing laxative effect and this substantiate the traditional use of the plant for its claimed indication.

## 1 Background

Constipation is a worldwide problem and it results in straining, infrequent of bowel movement, the occurrence of hard or lumpy stool, sensation of incomplete evacuation or blockage [1]. Many patients have physical and mental discomfort as a result of constipation, which can greatly disrupt their everyday lives and well-being [2].

Management of constipation with currently available medications is associated with side effects. Furthermore, some do not report satisfaction with their medication and others do not heal completely. For these reasons, developing safer and more effective treatments with sufficient efficacy, fewer side effects and reduced cost appears to be of significant importance. Medicinal plants, have shown to have a wide range of therapeutic characteristics, as a result, they have gotten a lot of attention as a supplement or as an alternative treatment option for constipation worldwide. Since, they are in some way more culturally acceptable and are cost effective, the use of medicinal plants in the treatment of constipation shows potential benefits [[3], [4], [5]].

**Fig. 1:**
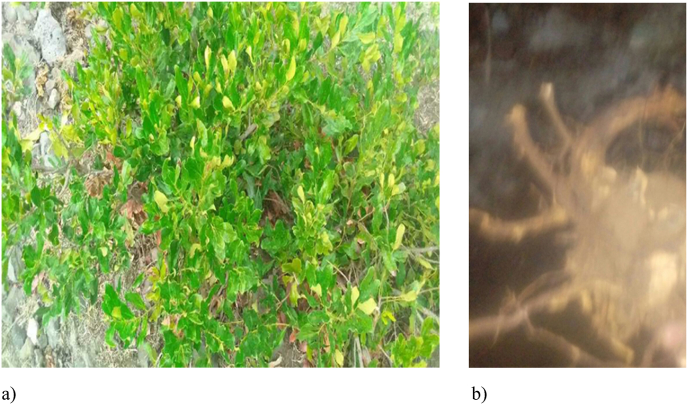
Photograph of *E. racemosa:* a) the whole plant, b) Parts used (root).

There are many plants, which are traditionally used for the treatment of constipation. *E. racemosa* L. is one of them (Figure-1). The plant material is prepared in the following way traditionally for the indicated condition. Initially, the root will be collected early in the morning. Thereafter, it will be boiled, and a full small teacup of the filtrate will be drunk before food. Even though *E. racemosa* L. has wider medicinal values including for laxative activity, its effect is not yet evaluated experimentally [6]. Therefore, the present study is aimed to investigate the laxative effects of aqueous root extracts of *E. racemosa* L. in mice using a different model in order to substantiate the claimed therapeutic effect. The findings of this study will give directions for the scientific community in conducting advanced research on molecular mechanisms and identifications of compounds that are responsible for laxative effect.

## 2 Materials and methods

### 2.1 Drugs and chemicals

The following drugs and chemicals were used in the experiment during the study period: loperamide (Medochemie Ltd, Limmasol, Cyprus), bisacodayl (Remedica Ltd. Limmasol, Cyprus), castor oil (Amman Pharmaceutical Industries Co, Jordan, activated charcoal (Sisco Research Laboratories Pvt. Ltd, India), ammonia, hydrochloric acid and ferric chloride (BDH Laboratory Supplies Ltd, England), acetic anhydride and Mayer‟s reagent (May and Baker Ltd, England), Dragendroff‟s reagent and sulfuric acid (Fisher Scientific, UK).

### 2.2 Plant materials

Both the leaves and the roots of *E. racemossa* L*. w*ere collected from Amhara region, North Shewa zone, Ensaro wereda, which is about 130 km north of Addis Ababa. It was collected on February 2022. To avoid change in physical appearance of the leaves and roots during transportation, the plant material was packed in plastic holding material. Thereafter, identification and authentication of the plant specimen was performed by a taxonomist Mr. Wege Abebe and a voucher specimen was deposited (voucher number of BM001) at the National Herbarium, College of Natural and Computational Sciences, Addis Ababa University for future reference.

### 2.3 Experimental animals

A total of 90 healthy swiss albino mice (body weight, 25–35 gm; age 6–8 weeks old) were used for the experiments. Female mice were used for acute toxicity study, and, randomly selected mice from either sex were used for the main study in all models. Majority of mice (60) were purchased from Ethiopian Public Health Institute and the remaining were obtained from the animal house of School of Pharmacy, College of Health Sciences, Addis Ababa University. Mice were maintained under standard conditions and fed on standard pellet diet and water ad libitum. They were then acclimatized to the environment for one week before commencement of the experiment. All animals were handled according to internationally accepted guidelines [7].

### 2.4 Extraction of the plant material

After the fresh root was cleaned and rinsed with tap water, it was chopped into small pieces and the size was further reduced by mortar and pestle. Then, the plant material was boiled in 1100 ml of water in 1:4 ratio for about 20 min [8,9]. Following this, the extract was allowed to cool. Thereafter, it was filtered twice using muslin cloth. The filtrate was finally concentrated using a hot oven at 37 °C. The dried extract was then weighed and transferred to vials until used. From 280 g of the plant material, 13 g of crude aqueous extracts of *E. racemosa* L. (percentage yield 4.64%) was obtained.

### 2.5 Acute toxicity

Acute oral toxicity was performed according to the Organization for Economic Cooperation and Development recommendation; five mice were fasted for 4 h before receiving 2000 mg/kg of aqueous root extracts of E. racemosa L. The mice were monitored for mortality and symptoms of toxicity for the first 30 min, then every 24 h for the next 14 days. The test was first performed initially, in a single mouse and this was followed by repeating the procedure on the remaining 4 mice as per guideline [7].

### 2.6 Induction of constipation

For inducing constipation, loperamide with a dose of 5 mg/kg was given for 6 days via the oral route. Likewise, the normal control group was given 10 ml/kg of distilled water for the same duration. Access to food and water was provided to all of the animals during the treatment period [10,11].

### 2.7 Grouping and dosing of animals

For determinations of laxative effect and GI motility test, mice constipated with loperamide were randomly divided in to 6 groups. One group (Group-1) which served as a normal control was also used and grouping was made in the following way: Group II (negative control) and group III (positive control) were treated with 10 ml/kg of distilled water and 0.25 mg/kg of bisacodyl (BIS0.25) respectively. The test groups (Group IV-VI) were treated with 100 mg/kg (ERAE100), 200 mg/kg (ERAE200) and 400 mg/kg (ERAE400) doses of *E. racemosa* aqueous root extract respectively.

For the third model (GI secretion test), mice were grouped in to five and grouping was made as follows: Group I (normal control) was received distilled water and group II (positive control) was given 0.5 ml castor. Groups III, IV and V (test groups) were given 100, 200, and 400 mg/kg of the crude extract, respectively. In all models, the normal control was treated with 10 ml/kg of distilled water. Moreover, in all cases mice were initially fasted for 18 h before they were treated with standard drug, extract or vehicle based on their grouping.

### 2.8 Laxative activity test in loperamide constipated mice

Prior to receiving the fifth dose, constipated mice were fasted for 18 h. After that, animals were kept in a separate cage with non-absorbable paper. The individual mice's fresh fecal pellets/wet feces were then counted and weighed in 2-h intervals throughout a 12-h period. The feces were then dried at room temperature for 24 h before being reweighed to assess the fecal water content. The formula below (formula-1) was used to compute the percent of fecal water content [12].

Formula-1 $$\%\;fecal\;water\;content=\frac{\;weight\;of\;wet\;fecal\;matter-weight\;of\;dry\;fecal\;matter}{weight\;of\;wet\;fecal\;matter}\;*100$$

### 2.9 Gastrointestinal motility test in loperamide constipated mice

A Gastric motility test was performed in loperamide-constipated mice as mentioned previously. Mice were fasted for 12 h before they were receiving the fifth dose of distilled water, 0.5 ml of castor oil and different doses of plant extract. Forty minutes later, each animal was treated with 0.3 ml of freshly prepared 5% aqueous suspension of charcoal meal orally. Then the animals were sacrificed. The abdomen was opened and small intestine from the pylorus to caecum was eviscerated after a half hr. Of charcoal meal administration. The distance traveled by the charcoal meal in the intestine, from the pylorus to the caecum was measured and the ratio of distance traveled by the charcoal meal to the total length of small intestine was calculated for each animal as follows (Formula-2) [13].

Formula 2 $$GI\;transit\;ratio\;\left(\%\right)=\frac{distance\;traveled\;by\;the\;charcoal\;meal}{Total\;length\;of\;small\;intestine}*100$$

### 2.10 Preliminary phytochemical analysis

The qualitative phytochemical investigation of the aqueous root extract of *E racemosa* was performed using standard tests with a slight modification [[14], [15], [16]].

#### 2.10.1 Test for terpenoids (salkowski test)

To 0.25 g of extract, 2 ml of chloroform was added. Then, 1.5 ml of concentrated sulfuric acid was added to form a layer. Finally, a reddish brown coloration of the interface indicates the presence of terpenoids.

#### 2.10.2 Test for saponins (honey comp test)

Five hundred mg of the extract was dissolved in a test tube containing 10 ml of DW and formations of honeycomb froth that persisted for half an hour was considered as positive for saponins.

#### 2.10.3 Test for tannins (ferric chloride test)

About 0.25 g of the extract was boiled in 10 ml of water and then filtered. Thereafter, 3 drops of 0.1% ferric chloride were added to the filtrate. The presence of tannins was confirmed by the formation of brown greenish or blue-black color.

#### 2.10.4 Test for steroids (liebermann burchard reaction)

One g of the extract was added to 10 ml of chloroform and then filtered. Two ml of acetic anhydride and concentrated H_2_SO4 were then added to the 2 ml of the extract. Formation of blue, greenish coloration indicates the presence of steroids.

#### 2.10.5 Test for alkaloids

The plant extract was mixed with 2% H2SO4 for 2 min. Then, it was filtered and few drops of reagents were added separately. Creamy-white colored precipitation (Mayer's reagent) appeared giving a positive result or a reddish-brown precipitate appeared (Wagner's reagent) which also confirms the presence of alkaloids in the extract.

#### 2.10.6 Test for flavonoids

Ten ml of ethyl acetate was added into a test tube having 0.25 g of the root extract and heated on a water bath for 3 min. The mixture was cooled and filtered. Then, 4 ml of the filtrate was taken and shaken with 1 ml of dilute ammonia solution. The layers were allowed to separate and the yellow color in the ammonia layer was taken as end point for the presence of flavonoids.

#### 2.10.7 Test for phenols

About 0.25 g of the root extract was treated with a few drops of 5% neutral ferric chloride solution; the appearance of a greenish color indicated the presence of phenols.

### 2.11 Data analysis

All statistical analyses were performed using international business machine of statistical package for the social Sciences, (IBM SPSS), version 25 for windows (SPSS inc, Chicago, Illinois, USA). Statistical differences between groups were analyzed by one-way analysis of variance (ANOVA) followed by Tukey post hoc test. Results were expressed as mean ± standard error mean (SEM) and P-values less than 0.05 were considered as statistically significant.

## 3 Results

### 3.1 Acute toxicity

The results of acute toxicity study revealed that the experimental plant extract is safe at a dose of 2000 mg/kg. Within 24 h since administrations of the aqueous root extract of E. racemose L*,* animals were found to tolerate the administered dose. There were no significant changes in behavior such as convulsion, breathing, restlessness, motor activity and change in skin color. Moreover, mortality was not recorded within 14 days of observations and lethal dose 50 (LD50) is assumed to be greater than 2000 mg/kg.

### 3.2 Laxative effects of the aqueous root extracts of *E. racemosa* L*.* In loperamide induced constipated mice

The middle (ERAE200) and highest doses (ERAE400) of plant extract produced significant fecal water content with percent fecal water content of 50.1 and 55.28 respectively (P < 0.05). Likewise, the standard drug (bisacodyl) produced an appreciable result in terms of percent fecal water content (57.69, P < 0.05). However, there was no recorded substantial mean weight of wet faces either from different doses of extract or from the standard drug (Table-1).

**Table 1:** Laxative effects of aqueous root extracts of *E. racemose* L. in loperamide induced constipated mice.

Groups	Weight of Animals	No of faces in 12 h	P-value	Weight of wet faces	P-value	Wright of dry faces	P-value	% fecal water content	P-value
NOC	24.0 ± 0.70	2.80 ± 0.73	1.00	0.063 ± 0.016	0.95	0.0478 ± 0.01	0.85	24.12 ± 4.23	0.87
NC	24.8 ± 0.37	2.2 ± 0.80	–	0.09 ± 0.029	–	0.083 ± 0.27	–	9.00 ± 3.4	–
BIS0.25	24.6 ± 0.50	7.2 ± 1.93	0.12	0.26 ± 0.15	0.001	0.11 ± 0.02	0.87	57.69 ± 10.76^b1^	0.03
ERAE100	31.8 ± 0.86	4.4 ± 1.28	0.74	0.059 ± 0.01	0.93	0.037 ± 0.082	0.68	35.93 ± 12.2	0.67
ERAE200	23.0 ± 0.70	6.6 ± 1.9	0.22	0.17 ± 0.04	0.25	0.085 ± 0.028	1.00	50.11 ± 12.8^b1^	0.04
ERAE400	29.6 ± 0.92	5.4 ± 0.74	0.55	0.26 ± 0.016	0.05	0.10 ± 0.18	0.98	55.28 ± 9.07^b1^	0.03

Each value represents mean ± S.E.M; n = 5 for each treatment; Analysis was performed by one way ANOVA; b, compared to negative control; NOC, normal control; NC, negative control; ERAE, *E. racemosa* L*.* aqueous extract; number followed by ERAE and BIS indicates dose in mg/kg. 1p < 0.05.

### 3.3 Effects of aqueous root extracts of *E. racemosa* L. On GI transit in loperamide induced constipated mice

The aqueous root extracts of *E. racemosa* L. produce a significant rise in intestinal motility (Table-2*).* The data revealed that the percentage increment in GI transit of charcoal meal was significant compared to the negative control with a transit ratio of 66.51% (p < 0.01), 80% and 93% (p < 0.001)) at a dose of 100 mg/kg, 200 mg/kg and 400 mg/kg respectively. In addition, all but ERAE100 of plant extract produced significant increment in intestinal motility compared to the normal control group. The standard drug's (bisacodayl) effect in increasing small intestine motility is also significantly higher compared to the normal (p < 0.01) and negative control group (p < 0.001).

**Table 2:** Effects of aqueous root extracts of *E. racemose* L. on GI transit in loperamide induced constipated mice.

Groups	Weight of the animal	Distance traveled by charcoal (cm)	P-value against NOC	P-value against NC	GI transit ratio (%)	P-value against NOC	P-value against NC
NOC	24.8 ± 0.66	16.6 ± 3.51	–	0.20	36.19 ± 1.73	–	0.47
NC	22.75 ± 0.54	7.80 ± 1.11	0.20	–	15.07 ± 2.32	0.47	–
BIS0.25	24.8 ± 0.86	27.36 ± 0.26^b3^	0.72	0.00	89.562 ± 0 3.34^a2b3^	0.001	0.00
ERAE100	26.0 ± 2.02	28.80 ± 2.39^a2b3^	0.03	0.00	66.51 ± 16.73^b2^	0.1313	0.00
ERAE200	32.20 ± 1.46	30.40 ± 4.36^a2b3^	0.01	0.00	79.82 ± 9.99^a1b3^	0.01	0.00
ERAE400	24.7 ± 1.06	32.56 ± 1.54^a3b3^	0.01	0.00	92.95 ± 2.28^a3b3^	0.001	0.00

Each value represents mean ± S.E.M; n = 5 for each treatment; Analysis was performed by one way ANOVA; a, compared to normal control; b, compared to negative control; NOC, normal control; NC, negative control; ERAE, *E. racemosa* L. aqueous extract; number followed by ERAE and BIS indicates dose in mg/kg. 1p < 0.05; 2p < 0.01; 3p < 0.001.

### 3.4 Effects of aqueous root extracts of *E. racemosa* L. On GI secretion in normal mice

**Fig. 2:**
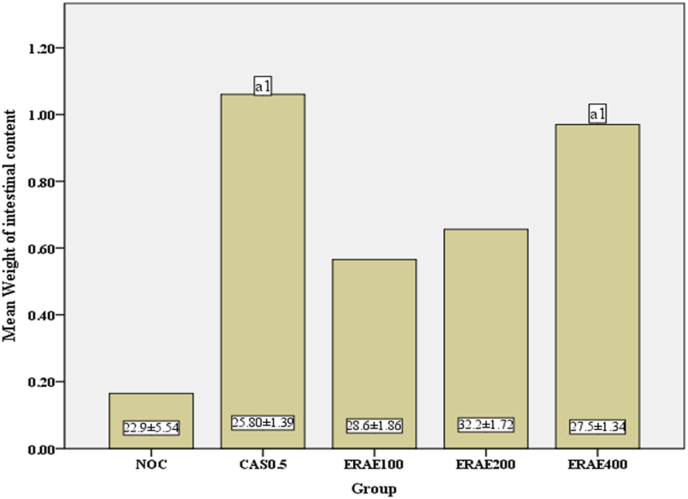
Effects of aqueous root extracts of *E. racemosa* L. on GI secretion in normal mice Each value represents mean ± S.E.M; n = 5 for each treatment; Values mentioned as mean ± S.E.M on each bars expresses weight of the experimental animas; Analysis was performed by one way ANOVA; a, compared to normal control; NOC, normal control; ERAE, *E. racemosa* L. aqueous extract; CAS, castor oil; number followed by ERAE and CAS indicates dose in mg/kg. 1p < 0.05.

When compared to the normal control group, the highest doses of plant extract (ERAE400) and the standard drug tend to produce a significant increase in intestinal fluid accumulation (P < 0.05), but the lowest and middle doses of aqueous extracts of the experimental plant failed to show a significant effect (Figure-2).

### 3.5 Phytochemical screening

The preliminary phytochemical screening of aqueous leaf extract of *E. racemosa* revealed the presence of terpenes, saponins, flavonoids, tannins and phenols (Table 3).

**Table 3:** Preliminary phytochemical screening of *E. racemosa* aqueous root extract.

Screened secondary metabolites	Test reagents/methods	Absence/presence
Terpenes	Salkowski test	Positive
Saponins	Honey comp test	Positive
Tannins	Ferric chloride test	Positive
Steroids	Liebermann Burchard reaction	Negative
Flavonoids	ethyl acetate	Positive
Alkaloids	Wagner's reagent	Negative
	Mayer's reagent	Negative
Phenols	Ferric chloride	Positive

## 4 Discussion

The experimental plant*, E. racemosa* L, is traditionally used for the treatment of gastritis, uterine prolapse, diarrhea, cataract, acne, chloasma, eczema and constipation [13]. It is rational to conduct this research because there is no other study, which aimed to confirm/support this claim, particularly, regarding its perceived use for the management of constipation. Therefore, the present study was directed to evaluate the laxative effects of the of the root extract of *E. racemosa* L. in swiss albino mice using three models. The plant extract was evaluated for its effect on the number of defecations and stool water content, fluid accumulation and intestinal transit ratio.

The use of loperamide as a constipation-inducing agent is well documented. Loperamide is an opioid agonist antidiarrheal agent that inhibits intestinal water secretion and colonic peristalsis [17]. The rationale behind the use of this drug was because previous studies have shown that administration of 2–5 mg/kg body weight of loperamide through oral and subcutaneous route for 3–7 days can successfully induce constipation in rodent [18,19].

In this experiment, oral administrations of aqueous root extracts of *E. racemosa* to the constipated mice was effective in increasing the percent of fecal water content which is one of the indications for the laxative effects of the experimental plant. Significantly, this effect was produced by the middle (ERAE200) and highest doses of plant extract (ERAE400) (P < 0.05). The percent fecal water content that was obtained from those doses was 50.11 and 55.28 respectively. The highest of such effect was observed in groups which the standard drug (bisacodyl) was administered (57.69, P < 0.05). Though the extract produced a significant in % of stool water content, it has been failed to produce an increased stool frequency over 12 h of measurement period. This might be due to the laxative effect of the plant extract is limited only in offsetting the constipation induced by loperamide without significantly increased frequency of deification. The plant extract possibly exerts the observed effect by disturbing the equilibrium between the absorption of water from the intestinal lumen via an active sodium transport and the secretion of water into the lumen [20].

The transit process of the entire GI tract reflected the overall GI motor activity. Measuring colonic transit time is useful in abdominal bloating, refractory irritable bowel syndrome and constipation. It also provides information about the identification and characterization of transit abnormalities and allows assessment of the severity of the problem as well as the response to therapy [21]. In the present study, 5% aqueous suspension of charcoal was used as a marker of the colonic movement. In this study, the aqueous root extracts of *E. racemosa* L. increased the intestinal motility which, in turn, enhances colonic peristalsis. Significant percentage increment in GI transit of charcoal meal was observed at all doses of plant extract with a transit ratio of 66.51% (p < 0.01), 80% and 93% (p < 0.001)) at doses of 100 mg/kg, 200 mg/kg and 400 mg/kg compared to the negative control group respectively. In addition, an apparent effect in the intestinal motility was observed with middle (P < 0.05) and the highest doses (p < 0.001) of plant extract compared to the normal control group. An increase in intestinal motility reduces the amount of time that intestinal contents remain in the intestine, which may shorten the time needed for the small intestine to absorb water and electrolytes. Therefore, the possible mechanism of the extract in this process may be enhancing the release of fluid thereby increasing intestinal secretion [22].

The final model (GI secretion) was used to examine the effect of *E. racemosa* L. extract on the small intestine fluid accumulation in normal mice. In this model, castor oil was used as a standard drug. Among the several mechanisms to explain the laxative effect of castor oil, one is the inhibition of intestinal Na+/K + -ATPase activity. Thus, reducing normal fluid absorption through activation of adenylate cyclase or mucosal cyclic adenosine monophosphate-mediated active secretion and nitric oxide release [23,24]. Moreover, castor oil promotes smooth muscle contraction via its metabolite, ricinoleic acid. This effect is mediated by activations of EP3 prostanoid receptors [25]. In this study highest doses of the aqueous root extracts of *E. racemosa* L*.* and the standard drug showed *a* significant increase in mean weight of intestinal content compared to the normal control group (p < 0.05). The significant rise in fluid content in the intestine by the highest dose of plant extract might be mediated by the same and previously mentioned mechanism of castor oil. In addition, laxative agents that increase water and electrolyte secretion in the intestinal lumen act by facilitating activation or expression of cystic fibrosis transmembrane conductance regulator (CFTR) or aquaporin [26]. Therefore, the effect of plant extract might be associated in augmentations of CFTR.

Overall, the observed laxative effects of the experimental plant could be due to the presence of secondary metabolites. The qualitative phytochemical screening test on the root extract of *E. racemosa* L. revealed the presence of terpenes, saponins, tannins, flavonoids and Phenols. Secondary metabolites such as such as alkaloids, phenols, tannins, saponins and flavonoids are reported to be responsible for the laxative, stimulant and GI propulsive activities of the plants. Moreover, some flavonoids like naringenin show increased secretion of Cl⁻ in the colonic epithelia of mice. Saponins have been reported to have smooth muscle contraction activity [1,27].

The current study has various public health implications. First, it is a major success to continue using traditional medicines once after it is evaluated and confirmed experimentally. Second using *E. racemosa* L. for it is claimed use as alternative medicine will help the general public in particular peoples leaving in the remote area who cannot afford and find modern medicine such as loperamide and magnesium hydroxide.

## 5 Conclusion

The present study reveals that the aqueous root extracts of *E. racemosa* exhibited laxative activity but the finding needs to be supported by further studies to disclose molecular mechanism of actions and identify the exact chemical constituents that are responsible for the laxative activity. The extract shows an increase in GI motility, intestinal fluid accumulation and a significant elevation in percent fecal water content. Therefore, this study validates the claim behind the use of *E. racemosa* L for. the treatment of constipation.

## 6 Limitations of the study

This study has some potential limitations. It has been conducted using only few models including laxative and GI transit effect on loperamide induced constipated mice as well as GI secretion effect of the plant extract. Therefore, it would be good if the study was further supported by other methods such as contraction effect of the extract on rabbit's intestine. Moreover for each model to avoid subject bias and cofounding factors each measurement should have been evaluated by other personnel than the authors. We have also conducted this study using only crude extract and investigations that help to understand the mechanism of the extract is not included.

## Ethics approval

The protocol was approved by institutional review board of the School of Pharmacy, College of Health sciences, Addis Ababa University with Reference no, ERB/SOP/451/14/2022.

## Consent for publication

Not applicable.

## Data availability

The datasets used for this study are available from the corresponding author on reasonable request.

## Funding

The study was done under the auspices of Addis Ababa University. The role of the funder on the research was providing animals and chemicals used in the experiment and.

## CRediT authorship contribution statement

**Akeberegn Gorems Ayele:** Data curation, Writing – original draft, Writing – review & editing. **Brooktawit Mulugeta:** Data curation, Writing – original draft, Writing – review & editing. **Yohannes Tsegyie Wondmkun:** Data curation, Writing – original draft, Writing – review & editing.

## Declaration of competing interest

The authors declare that they have no any financial and/or non-financial competing interests.
